# PP25 Artificial Intelligence In Healthcare Decision-Making: Addressing Challenges, Ethical Considerations, And Bias

**DOI:** 10.1017/S0266462324001983

**Published:** 2025-01-07

**Authors:** Rito Bergemann, Sugandh Sharma, Jackie Vanderpuye-Orle, Jean-Etienne Poirrier

## Abstract

**Introduction:**

Artificial intelligence (AI) is transforming healthcare decision-making, particularly in evidence evaluation and health technology assessment (HTA). This research explores challenges and ethical considerations associated with AI implementation, and biases. It highlights the need for diverse stakeholder perspectives and collaboration to ensure responsible AI use. Through transparency, accountability, and bias mitigation, AI has the potential to revolutionize decision-making and improve patient care while promoting equitable outcomes.

**Methods:**

Literature research was conducted, including peer-reviewed studies and grey literature, using the PEARL search strategy. Relevant articles from various databases and sources were screened and selected based on their alignment with the research objectives. The selected articles were then analyzed to identify key findings and insights related to the integration of AI in healthcare decision-making, ethical considerations, bias mitigation, and stakeholder perspectives.

**Results:**

The literature research revealed that AI in healthcare decision-making holds great promise. AI algorithms can efficiently analyze diverse healthcare data sources, improve evidence evaluation, and streamline decision-making processes. Ethical considerations, patient privacy and transparency are crucial. Bias in AI algorithms emerged as a significant challenge, requiring diverse and representative data, bias testing, and explainable AI. Stakeholder engagement plays a vital role in responsible AI implementation. Strategies for ongoing monitoring, collaboration, and training were identified to ensure fair and ethical decision-making in healthcare. The results emphasize the need for a balanced approach to harness the potential of AI while addressing its challenges.

**Conclusions:**

Integration of AI in healthcare decision-making offers promising opportunities but also presents challenges that need to be carefully navigated. By addressing ethical considerations and mitigating bias, AI can revolutionize decision-making, improve patient outcomes, and ensure the responsible and ethical use of AI in healthcare. The results provide valuable insights and recommendations for researchers working in the field of HTA.

